# Impact of Community-Based Food Interventions on Health, Well-being, and Social Connectedness of Older Adults: A Scoping Review

**DOI:** 10.1155/hsc/6677936

**Published:** 2025-10-25

**Authors:** Peter Jeff, Alison Benzimra, Jane L. Murphy, Lee-Ann Fenge, Camila Devis-Rozental, Sophia D. Amenyah

**Affiliations:** 1 Faculty of Health and Social Sciences, Bournemouth Gateway Building, Bournemouth BH8 8GP, UK; 2 United St Saviour’s Charity, 16 Crucifix Ln, London SE1 3JW, UK; 3 Office of the Vice Chancellor, Poole House, Bournemouth University, Talbot Campus, Fern Barrow, Poole BH12 5BB, UK; 4 Faculty of Health and Life Sciences, Northumbria University, Northumberland Building, College Street, Newcastle Upon Tyne NE1 8SG, UK

**Keywords:** community-based, health, nutrition, older adults, social connectedness, well-being

## Abstract

This scoping review examines the impact of community-based food interventions on older adults’ health, well-being, and social connectedness. As the global population ages, these interventions offer promising solutions to address health challenges older adults face, such as malnutrition, social isolation, and chronic diseases. This review finds that community-based food interventions effectively improve older adults’ dietary quality, physical health, and mental well-being, with more significant benefits observed when these interventions promote social bonding and foster a sense of community. Key factors contributing to success include combining multiple intervention components, such as nutritional education and physical activity. Offering culturally relevant food; incorporating interactive and sensory activities; embedding staff within interventions; including the involvement of experts; and clear goal-setting methods, such as SMART goals, are also crucial in driving behavior change and influencing the success of interventions. These elements foster a more personalized and holistic approach to health promotion. However, barriers such as limited time for social interaction, inadequate content delivery, challenges in accessibility and affordability, and limited food variety were identified. The insights from our review are significant for stakeholders integrating community-based food interventions into local healthcare systems, ultimately supporting healthy aging, improving the quality of life of community-based older adults, and reducing the burden on healthcare services, with economic benefits.

## 1 Introduction

Life expectancy and longevity are increasing globally; however, these gains in life expectancy do not necessarily translate to improvements in the quality of life (QOL). According to the World Health Organization (WHO) and United Nations (UN), the global population aged 60 years and over is projected to reach 1.4 billion by 2030, accounting for 17%–22% of the total global population [1–3]. In the United Kingdom, it is estimated that one in four people will be aged 65 or older by 2050 [4]. This demographic shift presents significant challenges for health and social care systems, which will face increased demand and costs. Increased longevity is correlated with a higher prevalence of noncommunicable diseases (NCDs), such as cardiovascular disease, diabetes, and obesity, which are linked to poor dietary habits and inadequate physical activity [5]. In addition, older adults face compounded risks of social isolation and loneliness, further impacting mortality and health outcomes [6]. An aging population requires novel strategies to enhance QOL, manage healthcare costs, and meet growing care demands. Food interventions have been shown to effectively reduce health risks and promote healthy aging, although reviews of evidence regarding community-based older adults are limited.

Promoting healthy aging involves addressing various factors that influence the overall well-being of older adults, including maintaining functional ability [5, 7]. Among the most significant are dietary patterns, social engagement, and physical activity—factors which are crucial for mitigating the risk of NCDs [8–10]. For older adults from low-income and socially disadvantaged backgrounds, where access to healthy food, social opportunities, and physical activity resources may be limited, these issues are more complex [11]. The UN’s strategies for promoting healthy aging emphasize establishing long-term care systems such as community-based service models. This involves collaboration with community organizations, sharing insights with stakeholders, and encouraging widespread adoption across countries. Such measures are essential for addressing the needs of an aging population [3]. Similarly, the WHO has called for urgent transformation of care services, advocating for personcentered, affordable, and accessible services that integrate health and social care. In this context, community-based food interventions have emerged as promising approaches to improve QOL and facilitate healthy aging while providing significant economic benefits. A 4-year evaluation of a community-based pilot model in the United Kingdom, which included services such as befriending, handyman call-outs, and transport assistance, has shown a return of £2.38 for every £1 invested, demonstrating the potential for cost-effectiveness in these interventions [12].

Older adults have an increased risk of NCDs and other health conditions, including malnutrition [13], sarcopenia [14], frailty [15], osteoporosis [16], and increased overall mortality [17]. They also experience a higher risk of social isolation and loneliness, which further compounds the increased risk of diseases. Before the pandemic, 1.4 million adults aged 50 and over in the United Kingdom reported frequently feeling lonely [18], and 29% of older adults were socially isolated [19]. The COVID-19 pandemic exacerbated these issues, with over 7.4 million British adults reporting that loneliness affected their well-being during the first lockdown [20]. More importantly, a recent meta-analysis found that loneliness itself may increase the risk of overall mortality by 26% [6].

Existing literature has identified several factors that influence the success of community-based food interventions, including establishing strong partnerships with local organizations, providing suitable environments for regularly sharing meals, sharing recipes between participants, the combination of theoretical education with practical sessions, interactivity of the intervention, and ongoing adaptability of the intervention. Addressing issues such as accessibility, transportation, and financial constraints and providing ongoing support and follow-up sessions are additional considerations that contribute to sustained success. However, a recent review found that, although older adults were included in some community-based interventions, their specific needs were often overlooked. This underscores the urgent need for more tailored approaches that consider this population’s unique behavioral and nutritional requirements.

To our knowledge, no previous scoping review has focused on food-based community interventions to improve older adults’ health, well-being, and social connectedness.

### 1.1 Study Objectives

The aim of the study was to synthesize the existing evidence on the impact of community-based food interventions on health, well-being, and social connectedness of older adults. It will also explore the mechanisms and strategies that contribute to the success of interventions and identify barriers to their implementation. The findings from this review will help inform future interventions and support policymakers in developing more effective, age-appropriate food interventions for promoting healthy aging. Specifically, this review will answer the following questions:

1. What types of community-based food interventions have been implemented to promote health, well-being, and social connectedness/engagement in older people?
2. Which strategies applied for these community-based food interventions have been successful or ineffective, and why?
3. What factors have contributed to the success or failure of these community-based food interventions for older people?

## 2 Materials and Methods

While community-based food models often represent system-level approaches to food security and nutrition, they can also be implemented as structured interventions in research settings. This scoping review explores both community-based food models and food interventions.

This review was conducted in accordance with the Joanna Briggs Institute (JBI) methodology for scoping reviews [21]. The findings are reported using the preferred reporting items for systematic reviews and meta-analyses extension for scoping reviews (PRISMA-ScR) [22]. The scoping review identified, retrieved, and evaluated information from peer-reviewed and gray literature articles that examined the impact of community-based food interventions on older people’s health, well-being, and social connectedness. Study protocol was registered with Protocols IO (DOI: https://doi.org/10.17504/protocols.io.5jyl82o46l2w/v1).

### 2.1 Search Strategy

Literature searches were conducted by two researchers (SDA, KP) in the following electronic databases: CINAHL complete, MEDLINE complete, Cochrane Library, PsycInfo, and SocINDEX using both medical subject headings and keywords, with searches in Scopus conducted using keywords. Additional searches of gray literature were conducted on Google, OpenDOAR, National Grey Literature Collection, PsycEXTRA (APA), the Social Science Research Network, and OpenAIRE.

The full search strategy (Supporting Table 1) combined terms related to older adults (e.g., older people and elderly), communities, community-based activities, food models and food interventions (e.g., food activities, gardening, cooking classes and social activities). Searches covered all studies published to date and were restricted to publications in English. The reference lists of included studies were also searched for potential references. The searches were conducted again before the final analysis, and any additional studies were retrieved and included.

### 2.2 Eligibility Criteria

Studies were included if they were randomized controlled trials (RCTs), intervention studies without randomization, quasi-experimental studies, and pre-post studies using both qualitative and quantitative data collection methods. Observational studies without an intervention were excluded.

#### 2.2.1 Inclusion Criteria

- Studies conducted in older adults aged 60 years and above.

#### 2.2.2 Exclusion Criteria

- Studies conducted in older people with specific health conditions or living in institutions or care homes.
- Interventions involving medication, supplementation, or targeted at changing the food environment or fiscal and regulatory policies.
- In vitro or nonhuman studies.

### 2.3 Data Extraction

Titles and abstracts of studies retrieved from the searches were independently screened by three reviewers (SDA, KP, and LB) to identify studies that met the inclusion criteria and were relevant to the aims of the review. Full-text articles identified as potentially meeting inclusion criteria were retrieved and reviewed using predesigned in/out forms to assess eligibility. Any disagreements over eligibility were resolved by a fourth reviewer, if necessary. The following data were extracted into a predesigned database: author, country, target group, study design, sample size, intervention description, length, location, outcomes related to health and well-being, dietary quality or intake, physical activity, QOL, and social engagement. Information on the effectiveness of the interventions and facilitators and barriers to their implementation were also extracted. Two reviewers (SDA and PJ) conducted data extraction independently, and the results were compared and discussed before final inclusion. Any disagreements were resolved by a third reviewer, if necessary.

### 2.4 Data Synthesis and Analysis

Data from included studies are presented in a narrative synthesis. Additional data on the effectiveness of the interventions were gathered from qualitative discussions in the included studies, which described how and why an intervention or parts of an intervention may or may not work and under what circumstances.

### 2.5 Quality Assessment

Quality assessment of included studies was carried out using the consolidated standards of reporting trials (CONSORT) checklist for RCTs [23] and a modification of the transparent reporting of evaluations with nonrandomized designs statement (TREND) for intervention studies without randomization [24].

## 3 Results

**Figure 1:**
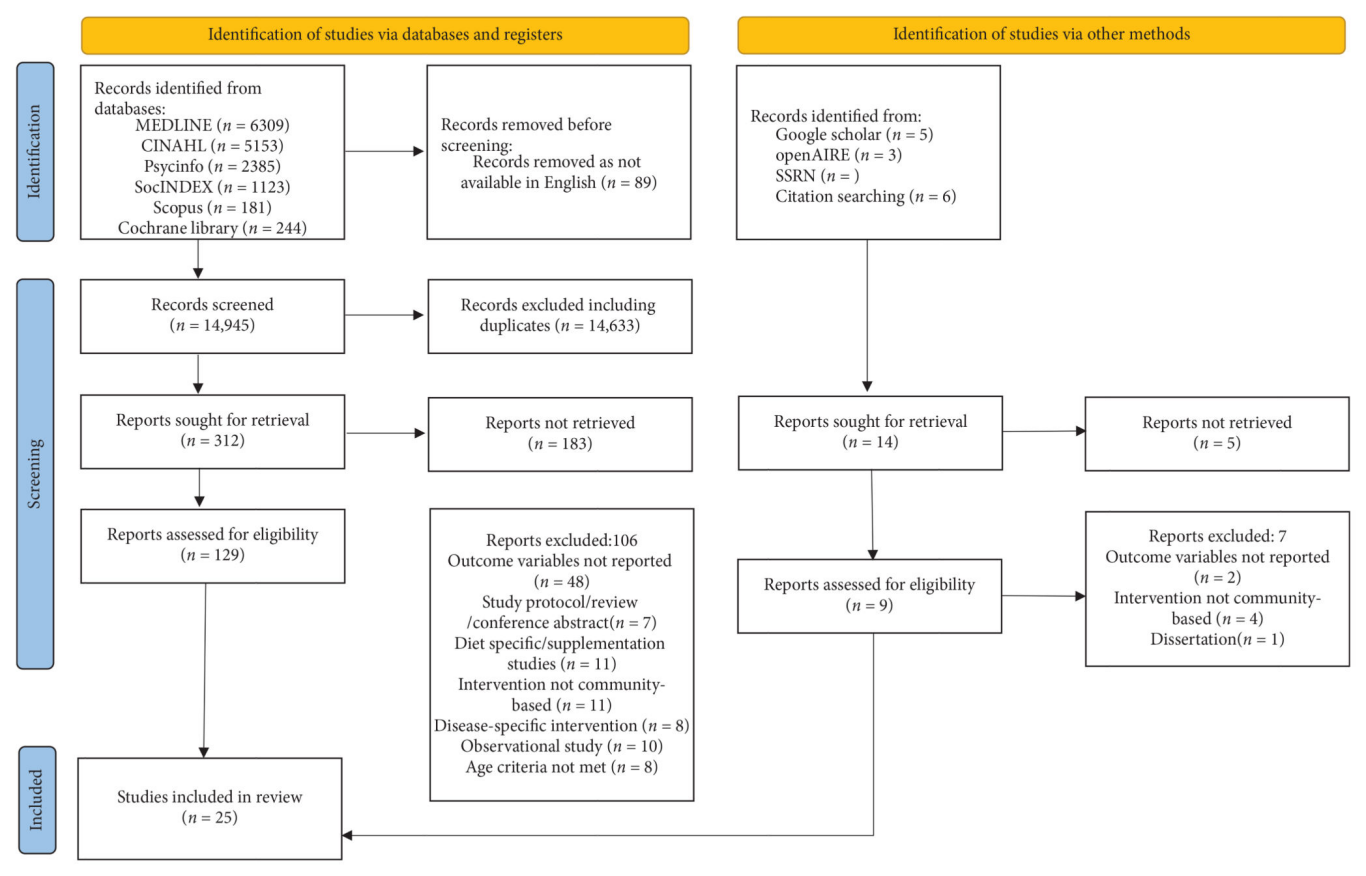
PRISMA flow diagram of study screening, selection, and inclusion.

A total of 25 studies meeting the inclusion criteria and deemed relevant to the aims of the study were included. Figure 1 illustrates the study screening, eligibility, and selection processes.

### 3.1 Characteristics of Included Studies

Tables 1 and 2 present the summary and main outcomes of the studies included in this review. The included studies were conducted across 11 countries: 10 studies from the USA (40%); 3 from Taiwan; 2 each from Japan, Singapore, and the United Kingdom; and 1 each from Norway, Italy, Ecuador, Iran, Brazil, and Mexico. The studies were conducted in the following locations: senior centers (13, 52%), community centers (9, 36%), gardens (2, 8%), unknown (2, 8%), local parks (1, 4%), community houses (1, 4%), health center auditoriums (1, 4%), senior apartments (1, 4%), retirement communities (1, 4%), nature reserves (1, 4%), national parks (1, 4%), research centers (1, 4%), agricultural farms (1, 4%), community common rooms (in low-income, disabled, or older adult apartments) (1, 4%), affordable housing sites (1, 4%), communal allotments (1, 4%), and senior citizen learning camps (1, 4%).

The total number of participants recruited across the 25 included studies was 3318, with a range of 10–614. Eleven (44%) of the studies were RCTs, 7 (28%) were quasiexperimental studies, 6 (24%) were pre-post studies, and there was 1 feasibility study (4%). Most (64%) of the included studies were multiple-component interventions (e.g., combining nutritional education and gardening) [27–33, 39–41, 43, 45, 47–49]. The remaining (36%) studies were single-component interventions involving only one distinct element (e.g., nutritional education or gardening alone). This category also included pre-post designs where a single intervention was applied before and after the study period without additional components [25, 26, 34–36, 38, 42, 44, 48].

Two broad intervention strategies were identified within the included studies as being either (a) nutritional education interventions aiming to increase nutritional awareness or knowledge [25–28, 30, 34–36, 38, 42–44, 46, 48] or (b) gardening or horticultural interventions aiming to improve mental and physical well-being [31, 33, 39–41, 45, 47, 49]. These strategies were subcategorized into: (i) fruit and vegetable intake [25, 26, 28, 43], (ii) nutritional awareness and knowledge [25, 34, 36, 38, 42], (iii) specific diet or food group education [30, 32, 37], (iv) nutritional education combined with physical activity [27, 28, 35, 39, 46, 48], (v) nutritional education combined with mental well-being [39, 43], (vi) social connectedness [27, 29, 31, 39, 41, 44, 45, 49], (vii) gardening for mental well-being [40, 41, 43, 44, 47], and (viii) gardening for both physical and mental well-being: [31, 45, 49].

### 3.2 Successful and Unsuccessful Intervention Strategies

Table 3 outlines successful and unsuccessful intervention strategies and the underlying mechanisms. Intervention strategies that proved successful include group-based interventions, multicomponent interventions, cooking and meal-sharing initiatives, interactive and sensory activities, goal-setting techniques, and the involvement of experts. Group interventions targeting fruit and vegetable consumption and improving nutritional awareness successfully promoted older adults’ health, well-being, and social connectedness [25–28, 30, 34–36, 38, 42–44, 46, 48]. These interventions led to notable outcomes, including improved nutritional status, enhanced nutritional knowledge, and increased social interaction because of the group setting. Multicomponent interventions [27–33, 39–41, 43, 45, 47–49], which combined different approaches, were generally more effective than single-component interventions, producing broader improvements across various health outcomes. Gardening interventions that included communal gardening and walking groups also proved particularly effective in enhancing mood and cognitive function, promoting social engagement, and reducing inflammation. Providing ample opportunities for older adults to cook and share meals and exchange recipes also significantly increased participation and the intake of healthy foods while facilitating social interaction [25, 26, 32, 36, 37].

Interactive and sensory activities significantly enhanced information retention and supported behavior change, including the use of songs and games [29, 30, 33, 37, 44, 47]. The use of structured models, such as SMART goals and the trans-theoretical model (TTM), enabled behavior change more effectively than approaches that were self-imposed or lacked clear goals [27, 43, 48, 50, 51]. Bringing in experts within group settings, such as chefs, dieticians, and gardeners, enhanced participant engagement by offering a personalized support contact and facilitating the development of key skills [25, 45].

Conversely, unsuccessful strategies include interventions that promoted nontraditional diets in countries where these dietary patterns were unfamiliar, single exposure to the intervention, and limited food variety both for consumption and growing produce. Interventions focusing on diets like DASH or Mediterranean faced challenges because of differences from traditional diets or the introduction of unfamiliar foods, highlighting the need to adapt specific diets to the target population more cautiously [30, 32]. Single-exposure interventions to increase fruit and vegetable intake showed no significant improvements, whereas repeated exposures effectively increased consumption [26]. Moreover, interventions that failed to offer a diverse selection of fruits and vegetables for either consumption or cultivation did not change consumption patterns and reduced participant satisfaction [26, 28, 45].

### 3.3 Mechanisms Underlying the Effectiveness of Implemented Interventions

Factors and mechanisms that contributed to successful interventions included conducting needs assessments, providing educational handouts, facilitating staff–participant relationships, providing sufficient time for familiarization before intervention, and using age-appropriate educational content. A needs assessment was deemed essential during the planning phase. This ensured that the interventions were customized to the unique needs and preferences of the participants, enhancing both engagement and the likelihood of positive outcomes [25, 33, 36, 37]. Practical resources including the provision of nutrition education materials to participants was important in promoting healthier dietary choices at home and encouraging continued participation [27, 28, 34, 37]. Strong staff–participant relationships played a significant role in the success of implemented interventions, facilitating the delivery of content and social cohesion within the community and increasing participant engagement and retention [33, 36, 47]. Providing sufficient time and opportunities for familiarization between participants also emerged as an essential factor [25]. Age-appropriate educational content and materials tailored to participants’ cognitive levels facilitated understanding and engagement and improved effectiveness by ensuring that the information provided was accessible and suitable for the target population. Interventions that reviewed previous educational content during subsequent sessions improved knowledge retention and increased long-term adherence [34, 37, 44].

Conversely, factors that contributed to poor delivery or failure of some interventions included high dropout rates, complex presentation content, limited food variety, seasonal constraints, unsuitable questionnaires, and affordability issues relating to the cost of produce. As older adults faced a higher risk of NCDs, many interventions, particularly long-term interventions, suffered high dropout rates, limiting evaluation upon follow-up [28, 31]. Presentations that were overly complex or difficult to read also detracted from the overall effectiveness of the interventions, suggesting a refinement of guidance for older adults, as it was found that older adults prefer educational methods that do not involve PowerPoint [25, 37].

Selection bias during recruitment limited the diversity of cohorts, as many recruited participants frequent the locations where recruitment flyers were posted [35, 36], It is important to note that the demographics of the included studies were predominantly white and female. Seasonal constraints hindered participation, highlighting the need for interventions to design both indoor and outdoor activity options to maintain year-round engagement [35]. Interventions should use suitably designed questionnaires, as one study raised concerns surrounding complex language and cultural biases [33]. Lastly, affordability was a significant barrier to engagement. Participants struggled to afford fresh ingredients, and the intervention failed to affect their perceptions [34].

## 4 Discussion

This scoping review aimed to synthesize the existing evidence on community-based food interventions that promote health, well-being, and social connectedness among older adults. Our findings enhance the understanding of strategies and barriers that influence the success and effectiveness of these interventions. We provide novel insights on how community-based food interventions and models can be optimally designed to support healthy aging within older communities and the factors influencing them. These contributions have significant implications for creating effective and engaging interventions that can be integrated into healthcare systems, ultimately improving the overall wellbeing of older adults and reducing the burden on medical systems. The included studies encompass various models of community-based food interventions, primarily from the USA. The majority of studies focused on nutrition education as a strategy to improve intake of healthy diets [25–28, 30, 32, 34–38, 42–44, 46, 48, 51], increasing fruit and vegetable intake [26, 28, 34, 43], increasing physical activity [27, 29, 35, 41, 45–49, 51], or using gardening to improve mental and physical health [29, 31, 33, 40, 41, 45, 47, 49]. Only five studies measured social connectedness or engagement [27–29, 31, 41], although it was mentioned incidentally in others. Most interventions enhancing health, well-being, and social connectedness demonstrated statistically significant positive outcomes. These outcomes included improved blood pressure, waist-to-hip ratio, QOL, cognitive function, inflammation levels, psychological well-being and mood, perceived stress, social engagement, nutritional status, and knowledge of healthy behaviors and dietary patterns.

Research indicates multiple benefits of gardening and horticultural activities, and accessibility of these types of activities is improved when waist-height garden beds are utilized [45]. Gardening is widely recognized for promoting overall health, particularly in urban areas where access to green spaces is limited. It enhances well-being and provides opportunities for social interaction and cohesion. It increases physical activity through growing produce and improves nutritional quality with the increased consumption of plant foods. In addition, gardening has been increasingly shown to alleviate depression and anxiety, boost mood and cognitive function, and enhance QOL. When set in communal spaces, it fosters social connections, reduces isolation, and nurtures a sense of belonging [50, 52, 53]. Similar to other health promotion strategies, the effectiveness of gardening interventions depends on the design and how it is tailored to meet the specific needs of older adults within communities. Interventions undertaken within established communities, particularly those involving older adults in shared activities, are particularly effective [54].

Implementing food interventions within established communities engages individuals and provides adaptable frameworks upon which to build. Social connectedness plays a critical role in the health of older adults, and the lack of positive social relationships is a significant risk factor for poor mental and physical well-being. Those who experience social isolation often perceive more social threats, but these feelings can also motivate them to renew social connections. If an effective model is introduced, isolated individuals may be more likely to engage with their community [55]. Interventions encouraging social interaction and food-based activities, such as shared meals and food-tasting sessions, offer valuable opportunities for social engagement. Group-based activities improve mental well-being, give a sense of belonging, and enhance nutritional status. In contrast, eating alone is associated with adverse outcomes, including depression, decreased social support, and malnutrition [56, 57].

The success of community-based food interventions is influenced by a combination of demographic, environmental, individual, and social factors [57, 58]. Societal factors, such as the size of older adults’ social networks, support, and availability of social spaces, underpin the success of interventions. Demographic factors, such as age, socioeconomic status, and cultural background, can affect how individuals respond to or participate in interventions. Older adults from lower socioeconomic backgrounds may face additional barriers, such as affordability. Individual factors, including participants’ motivation, health status, and prior knowledge of healthy lifestyle behaviors, also impact engagement and effectiveness and influences the planning of educational content. Environmental factors, including settings that facilitate social interaction, such as community gardens and congregate meal sites, are essential for facilitating social connectedness and creating social networks that support long-term engagement. Interventions encompassing problem-solving, sensory, and interactive activities enhanced cognitive outcomes among older adults. This review indicates that these components positively influence nutrition knowledge and mental well-being. Declines in sensory abilities, especially vision and hearing, are often linked to cognitive decline in older adults. As a result, sensory-based activities are particularly effective in promoting behavior change and supporting mental health, as they can stimulate cognitive function and boost emotional well-being [58, 59].

Furthermore, multicomponent interventions generally yield better outcomes than single-component approaches, aligning with broader research [60, 61]. When multiple factors such as social participation, physical activity, and nutritional education are addressed in one intervention, they tend to be more effective than focusing on a single aspect, based on outcomes like cognitive health, waist-to-hip ratio, and dietary variety [27, 35, 48, 61, 62]. Interventions that combine strategies with extra elements, such as cooking workshops or mental well-being services, show particular promise [63]. These comprehensive strategies are more likely to produce long-term benefits by simultaneously targeting multiple aspects of health.

Research gaps include the limited use of digital tools, with only one study utilizing mobile apps for nutrition education. As technology integration in health services continues to grow, it would be beneficial to incorporate digital tools into future interventions. There was also a lack of focus on studies in underserved populations, including older adults from low-income and ethnic minority populations [34, 45]. Social connectedness was often observed as an incidental finding rather than a primary outcome, although generally positive results were observed. Furthermore, selection bias during recruitment limited the diversity of the participant cohorts, as many individuals frequented the locations where recruitment flyers were displayed.

The main challenges associated with less successful interventions included insufficient time for participants to develop meaningful connections, a lack of culturally appropriate food options or limited food variety, and seasonal factors that limited participation. Limited opportunities for social interaction may have reduced engagement and the sense of community that is often critical for the success of group-based interventions. Similarly, the absence of culturally appropriate food options or restricted variety could have affected participants’ satisfaction, adherence, and overall nutritional outcomes, particularly for individuals from diverse backgrounds. Seasonal factors, such as adverse weather or holiday periods, further restricted attendance and consistency, limiting the interventions’ effectiveness. Additional difficulties arose in designing content that was both suitable and engaging for participants, highlighting the challenge of balancing educational objectives with participants’ interests, needs, and literacy levels. Practical barriers relating to accessibility and affordability also played a significant role, potentially excluding individuals with mobility limitations, financial constraints, or other socioeconomic disadvantages. Collectively, these challenges underscore the complexity of delivering interventions in real-world settings and the need for careful planning, cultural sensitivity, and flexible delivery strategies to maximize reach, participation, and positive outcomes.

This scoping review has both strengths and important limitations. Using data from both peer-reviewed publications and gray literature, it provides a comprehensive analysis and examines various health outcomes, including weight, waist circumference, mental well-being, cognitive function, and social connectedness in older adults. The review encompasses a wide range of health outcomes and a comprehensive overview of the potential impacts of community-based interventions on older adults.

However, there are some limitations to consider. The included studies varied considerably in methodological rigor and data analysis approaches, particularly in the measures employed to assess outcomes and the diverse range of questionnaires used, many of which were study-specific rather than standardized. A further limitation is the reliance in many cases on subjective self-reported health measures, which may introduce reporting bias and reduce the reliability of findings. In addition, the study designs were often less robust, frequently nonrandomized, or lacking appropriate control groups, which reflects the inherent challenges of evaluating this type of complex intervention in real-world settings. Another important consideration is the limited diversity of the populations studied. Many of the Health & Social Care in the Community included studies were conducted with relatively homogenous samples in terms of ethnicity, socioeconomic back-ground, and geographical setting. This lack of diversity restricts the generalizability of findings and raises questions about the extent to which the reported outcomes can be applied across wider, more heterogeneous populations. Future research would therefore benefit from adopting more rigorous study designs, incorporating both objective and subjective outcome measures. In addition, ensuring greater diversity in the populations of older adults included in community-based interventions will support more inclusive research and the development of evidence-based policies that benefit a wider range of older people, particularly those from underserved communities.

## 5 Conclusions

Community-based food interventions included in this review successfully improved dietary quality, social connections, and overall mental and physical well-being of older adults. These benefits were more significant when interventions promoted social bonding and a sense of belonging within communities. Effective interventions combined multiple elements, including nutritional education and physical activities. Engaging activities, such as sensory and interactive experiences, including gardening, and experts’ involvement, were crucial in encouraging participation and facilitating behavior change. Educational materials and surveys should be age- and culturally appropriate and presented in clear, simple language, rather than theoretical, to improve learning and information retention.

Future interventions should prioritize a group-based multicomponent approach to maximize the likelihood of success. This should be supported by comprehensive needs assessments of the targeted populations and expectations of further adaptation, collaboration with local community stakeholders, and including older adults as co-designers. Incorporating appropriate technology and addressing accessibility and affordability concerns are also vital for achieving meaningful and sustainable outcomes. Our review findings will significantly benefit stakeholders in developing community-based food models by enhancing long-term success, fostering social connections, and ensuring smooth integration with current social networks and healthcare systems.

## Supplementary Material

Supporting Information

## Figures and Tables

**Table 1:** Study characteristics and main outcome of included studies.

Author	Country	Study design	Age (average/range)	Sample size	Type of intervention	Location	Duration (weeks)	Main outcome
Aakre et al. 2023 [25]	Norway	Feasibility study	Not reported	16	Nutritional education with mobile app.	Not reported.	2 days	↑ Increased awareness of nutrition, understanding the importance of shared meals, and enhanced critical thinking about advertisements.
Appleton, 2013 [26]	UK	RCT	74	95	Exposure to fruit intake.	Not reported.	5	↑ Increased fruit intake (*p* < 0.01) in participants with initially low fruit consumption. No change in preference for fruit.
Barnett and Zeng 2022 [27]	USA	RCT	73	292	Education program - healthy eating for successful living in older adults (HESL).	Senior centers, housing authorities, churches, assisted living facilities, libraries.	6	↓ Decreased waist-to-hip ratio (*p* < 0.05), ↑ increased healthy behavior index (*p* < 0.001). ↑ Increased self-reported fruit and vegetable intake. No change in BMI or waist circumference. No change in quality of life, physical activity, or social connectedness scores.
Brewer et al. 2016 [28]	USA	Quasi-experimental.	75	35	Nutrition education.	Senior centers.	16	↑ Increased fruit and vegetable intake and frequency (*p* = 0.03), improved phytochemical knowledge, and ↑ increased sharing of knowledge (*p* = 0.04).
Chan et al. 2022 [29]	Taiwan	Quasiexperimental.	65−82	86	Horticultural.	Community centers.	8	↑ Increased WHOQOL-BREF physical health score (*p* = 0.008), quality of life (*p* = 0.006), working memory (*p* = 0.014), social relationships (*p* = 0.006), and psychological health (p< 0.001). ↓ Decreased perceived stress (*p* < 0.001)
Chou et al. 2021 [30]	Taiwan	RCT	65−90	80	Nutrition education.	Community service centers.	8	↑ Increased Mediterranean dietary behavior scores (*p* < 0.001), ↑ increased objective cognitive function (*p* < 0.001). ↓ Decreased subjective cognitive function (*p* < 0.001).
Gagliardi et al. 2019 [31]	Italy	Pre-post intervention.	73	112	Horticultural.	Agricultural farms.	52	No change in WHOQOL-AGE (*p* = 0.413). ↑ Increased contact with friends (*p* = 0.001) and relatives (*p* = 0.009), frequency of gardening (*p* = 0.001), and number of leisure activities (*p* = 0.002).
Hashemi et al. 2022 [32]	USA	Pre-post intervention.	73	94	Nutritional education	Senior centers	26	↓ Decreased systolic blood pressure (-4.41 mmHg) (*p* = 0.07). ↓ Decreased self-reported systolic blood pressure (-6.9 mmHg) (*p* = 0.004).
Heliker et al. 2000 [33]	USA	Quasi-experimental.	74	24	Horticultural.	Senior centers, botanical gardens.	18	↑ Enhanced perceived well-being (*p* < 0.000). No change in physical subscale, life attitude profile, or SOMP-M.
Hersey et al. 2015 [34]	USA	Quasi-experimental.	60−80	614	Nutritional education, focusing on fruit and vegetable intake.	Senior centers.	4	↑ Increased fruit consumption (*p* < 0.05), vegetable consumption (*p* < 0.01), and fruit and vegetable consumption (*p* < 0.01). ↑ Increased likelihood of health discussions with healthcare professionals (*p* < 0.05) and family/friends (*p* < 0.01).
Kimura et al. 2013 [35]	Tokyo	RCT	65−90	92	Nutrition education with physical activity.	Community centers	60	↑ Increased dietary variety scores (*p* = 0.001), ↑ Increased self-rated health (*p* = 0.039). No change in TMIG scores.
Klinedinst, 2005 [36]	USA	Pre-post intervention	60−92	25	Nutrition education	Community common room	3	↑ Increased nutrition knowledge based on the health belief model.
MacNab et al. 2017 [37]	USA	Pre-post intervention	60−81+	157	Nutrition education	Senior apartments, retirement communities, senior centers	3	↑ Increased knowledge of whole grains (*p* < 0.001), total grain intake frequency (p ≤ 0.001). ↑ Increased liking of whole grains (p = 0.019) and 59.2% intention to eat more whole grains.
McClelland et al. 2015 [38]	USA	RCT	79	458	Nutrition education using song	Congregated nutrition sites	1 day	↑ Increased nutrition knowledge (*p* < 0.001).
Mendoza et al. 2015 [39]	Mexico	RCT	70	64	Nutrition education with physical activity	Senior centers	8	↑ Increased nutritional status (*p* < 0.05). ↓ Decreased risk of falls (*p* < 0.05).
Milligan et al. 2004 [40]	UK	Pre-post intervention	65−79	30	Gardening	Communal allotments	39	↑ Increased sense of achievement, satisfaction, and esthetic pleasure.
Ng et al. 2018 [41]	Singapore	RCT	67	59	Gardening and walking	Parks, gardens, nature reserves	26	↓ Decreased IL-6 levels from baseline to 6 months (−0.23 pg/ml) (*p* = 0.02), a decreasing trend in BDNF levels (−101.45 pg/mL) (*p* = 0.07). ↑ Increased anxiety scores (*p* < 0.001). ↑ Increased psychological well-being (*p* = 0.001). No change in MoCA scores. No change in IL-1β, cortisol, DHEA, or hs-CRP.
Ortiz et al. 2023 [42]	Ecuador	Quasi-experimental	74	109	Nutrition education	Community houses, health center auditoriums	12	↑ Increased knowledge of healthy eating based on a designed questionnaire (*p* < 0.05).
Salehi et al. 2011 [43]	Iran	Quasi-experimental	64	400	Nutrition education	Senior centers	4	↑ Increased fruit and vegetable intake (*p* = 0.001), perceived benefits (*p* < 0.001), and self-efficacy (*p* < 0.001). ↓ Decreased perceived barriers (*p* < 0.001). Increase in the number of participants in the intervention group moved from the pre-contemplation phase to the contemplation/preparation and action/maintenance stages (χ^2^ = 233.7, p< 0.00001), and from contemplation/preparation to action/maintenance stages (χ^2^ = 8.1, *p* = 0.004) (stages of the change model).
Scariot et al. 2023 [44]	Brazil	RCT	69	36	Nutritional education, psychopedagogical program (PP), & culinary workshop program (CWP)	Community centers	12	↑ Increased nutrition knowledge in diabetes (*p* = 0.033).
Strout et al. 2017 [45]	USA	Pre-post intervention	77	10	Horticultural	Affordable housing sites	17	↑ Increased self-rated health and nutritional status, frequency of fruit and vegetable intake, hydration, and mini-mental state examination scores.
Towne et al. 2018 [46]	Texas	Quasi-experimental	73	260	Nutrition education with physical activity	Community centers, senior centers	10	↑ Increased MVPA in participants with less than 150 min of MVPA at baseline (*p* = 0.0210). f MVPA of over 160 min across all participants (*p* < 0.0001).
Tu et al. 2020 [47]	Taiwan	Pre-post intervention	68	27	Horticultural	Senior citizens learning camp	1	No change in systolic and diastolic blood pressure post-activity. ↓ Decreased salivary amylase activity after activities: leaf prints (*p* = 0.012), herb tasting and smelling (*p* = 0.010), and Kokedama (*p* < 0.001). ↑ Increased POMS scores post-activities: leaf prints (*p* < 0.001), herb tasting and smelling (*p* < 0.001), Kokedama (*p* = 0.035), and grass doll (*p* = 0.046).
Uemura et al. 2018 [48]	Japan	RCT	72	84	Nutrition education with physical activity	Community centers	24	↑ Increased HLS-14 (*p* = 0.03), ↑ FFQ scores (*p* = 0.001), and ↑ dietary variety scores (*p* = 0.04). ↑ Physical activity levels (*p* = 0.01), ↑ walking speed (*p* < 0.001), and ↑ steps per day (*p* < 0.001). No change in HLS-EU-Q16 (*p* = 0.18). No change in grip strength (*p* = 0.09).
Wong et al. 2021 [49]	Singapore	RCT	67	59	Horticultural	Research centers, national parks	26	↓ Decreased IL-6 levels

Note: All studies involved male and female participants, except Scariot et al., 2023 which only recruited females. Abbreviations: BMI, body mass index; DHEA, dehydroepiandrosterone; FFQ, food frequency questionnaire; HLS-14, health literacy scale 14; HLS-EU-Q16, health literacy survey European questionnaire16; hs-CRP, high-sensitivity c-reactive protein; IL-1 β, interleukin-1 beta; IL-6, interleukin-6; MOCA, Montreal cognitive assessment; MVPA, moderate-to-vigorous physical activity; POMS, profile of mood states; SOMP-M, social and occupational medicine profile; TMIG, Tokyo metropolitan institute of gerontology index of competence; WHOQOL-AGE, World Health Organization quality of life age; WHOQOL-BREF, World Health Organization quality of life brief; ZUNG SAS, Zung self-rating anxiety scale.

**Table 2:** Impact of interventions grouped according to outcomes relating to health, well-being, and social connectedness.

Author	Health	Well-being	Social connectedness
Aakre et al. 2023 [25]	Increased awareness of nutrition and consumption of spices.	Nutritional awareness and critical evaluation of advertisements increased.	Increased awareness of the importance of shared meals and opportunities for socialization.
Appleton, 2013 [26]	Increased fruit intake.	Not reported	Increased socialization opportunities.
Barnett and Zeng 2022 [27]	Improved health behaviors and increased fruit and vegetable intake.	No change in physical activity.	No changes in quality of life and social connectedness.
Brewer et al. 2016 [28]	Increased fruit and vegetable consumption.	Increased engagement in healthy fruit and vegetable behaviors.	Increased sharing of new knowledge with friends and family.
Chan et al. 2022 [29]	Improved physical health.	Improved quality of life and working memory and decreased perceived stress.	Improved psychological and social well-being.
Chou et al. 2021 [30]	Improved dietary behavior.	Improved objective cognitive function and decreased subjective cognitive function.	Not reported
Gagliardi et al. 2019 [31]	Not reported	No change in quality of life.	Increased social contact, home gardening participation, and overall engagement in leisure activities.
Hashemi et al. 2022 [32]	Improved blood pressure	Not reported	Not reported
Heliker et al. 2000 [33]	Not reported	Improved psychological well-being and garden skills, no changes in physical well-being, life attitude.	Enjoyment of social interactions with investigators and peers.
Kimura et al. 2013 [35]	Increased intake of several food groups, overall dietary variety, food frequency, and self-rated health.	Improved intellectual activity and self-maintenance.	Improved social roles.
Klinedinst, 2005 [36]	Enhanced understanding of nutrition.	Not reported	Improved social interaction.
MacNab et al. 2017 [37]	Increased knowledge and consumption of whole grains.	Increased liking and knowledge of whole grains.	Not reported
McClelland et al. 2015 [38]	Improved nutritional knowledge.	Not reported	Not reported
Milligan et al. 2004 [40]	Not reported.	Participants reported an improved sense of achievement, satisfaction, and esthetic pleasure.	Allotments (community gardens) were found to contribute to the social inclusion of older adults.
Ng et al. 2018 [41]	Horticulture therapy was associated with decreased inflammation.	No change in cognitive function.	Improved psychological well-being.
Ortiz Segarra et al. 2023 [42]	Increased knowledge of healthy eating.	Not reported	Not reported
Salehi et al. 2011 [43]	Increased daily fruit and vegetable intake and supported participants’ progression through the stages of change model toward healthier eating behaviors.	Increased perceived benefits and self-efficacy while reducing perceived barriers to healthy eating.	Not reported
Scariot et al. 2023 [44]	No change in general nutrition knowledge, but diabetes-specific nutritional knowledge improved.	Not reported	Not reported
Strout et al. 2017 [45]	Improved self-rated health and nutritional status after the intervention. Increases in frequency of fruit and vegetable intake, along with better hydration.	Improved cognitive function, as shown by increased MMSE scores.	The intervention was enjoyable and rewarding, fostering social connections through sharing surplus food, recipes, and knowledge.
Towne et al. 2018 [46]	Increased physical activity.	Not reported	Not reported
Tu et al. 2020 [47]	Reduced physiological stress responses following participation in these activities.	Positive effects on mood and emotional well-being after participation in horticultural activities.	Not reported
Uemura et al. 2018 [48]	Improved health literacy and dietary variety.	Improved cognitive function, physical function, and physical activity levels, including increases in walking speed and daily steps (around 60% more). There was no change in grip strength.	Not reported
Wong et al. 2021 [49]	Decrease in inflammation biomarkers.	Not reported	Not reported
Hersey et al. 2015 [34]	Improved dietary intake.	Participants more likely to discuss fruit and vegetable choices with healthcare providers and with friends or family.	Not reported
Mendoza-Ruvalcaba and Arias-Merino 2015 [39]	Improved nutritional status.	Reduced risk of falls post-intervention.	Not reported

**Table 3:** Factors contributing to the success or unsuccessful outcomes of included studies.

Intervention strategy	Factors underlying successful interventions	Factors underlying unsuccessful interventions
Nutritional education [25, 27, 28, 32, 34, 35, 37–39, 42–44, 46, 48]	Meals were prepared collaboratively with participants, enhancing engagement. Educational materials focused on phytochemicals, “eat the rainbow” messages, and the Mediterranean diet increased nutritional knowledge. Game-based and hands-on learning, including tasting sessions, reinforced learning, and retention. Group-based activities and familiar environments promoted social interaction among participants, families, and staff. Adaptable, age-appropriate materials and at-home activity options improved accessibility and participation. Staff involvement and structured approaches supported self-efficacy.	Content that was too general and not personalized. Overly theoretical language. Insufficient follow-up. Seasonal constraints affected engagement. Presentation style was not engaging. Significant concern about cost of fruits and vegetables.
Provision of healthy food options, including fruits and vegetables [26, 28]	Repeated exposure to healthy food options. Meal provision encouraged participation.	
Gardening and horticultural interventions [31, 33, 40,41,45, 47–49]	Social interaction and physical activity were promoted in a farm setting. Open-ended interviews helped build connections, and gardening evoked positive memories. Communal gardening encouraged social interaction and improved social well-being. Waist-height garden beds and expert guidance facilitated participation and social interaction. Group-based engaging activities stimulated multiple senses and promoted social interaction, with instructor involvement having a strong impact. Long-term group-based activities emphasized gardening for physical activity and mental health, sustaining social interaction.	Complex language and cultural biases. Variety of produce provided was limited.

## Data Availability

Data sharing is not applicable to this article as no datasets were generated or analyzed during the current study.
