# Supplementary material for: Impact of Community-Based Food Interventions on Health, Well-being, and Social Connectedness of Older Adults: A Scoping Review
Source: Health Soc Care Community. Author manuscript; Available in PMC 2025 Dec 4. (PMC7618437; doi:10.1155/hsc/6677936)
Supplement: Supporting Information [file EMS210843-supplement-Supporting_Information.docx]

**Supplementary Table S1: Systematic search strategy**

Systematic searches were conducted in 4 databases including the Cochrane library, Medline, SocIndex, Cumulative Index to Nursing and Allied Health Literature (CINAHL), Scopus, and PsychInfo The search strategies for all databases are detailed below:

1. **Cochrane Library**

ID Search Hits

#1 MeSH descriptor: [Geriatrics] explode all trees 400

#2 MeSH descriptor: [Aged] explode all trees 255877

#3 MeSH descriptor: [Aging] explode all trees 5081

#4 MeSH descriptor: [Healthy Aging] explode all trees 116

#5 older population 16511

#6 older adults 27392

#7 older person 7372

#8 elderly 60737

#9 geriatrics 6573

#10 senior citizens 169

#11 senior 5578

#12 aged over 60 30088

#13 aged over 65 22884

#14 ageing 24011

#15 aging 24008

#16 healthy aging 5374

#17 1 or 2 or 3 or 4 or 5 or 6 or 7 or 8 or 9 or 10 or 11 or 12 or 13 or 14 or 15 or 16 2045282

#18 MeSH descriptor: [Food] explode all trees 45523

#19 MeSH descriptor: [Cooking] explode all trees 495

#20 MeSH descriptor: [Food] explode all trees 45523

#21 MeSH descriptor: [Drinking] explode all trees 1142

#22 MeSH descriptor: [Gardening] explode all trees 59

#23 MeSH descriptor: [Gardens] explode all trees 17

#24 MeSH descriptor: [Diet, Healthy] explode all trees 790

#25 food model 6318

#26 nutrition model 6110

#27 food guide 1319

#28 food club 138

#29 lunch club 20

#30 cooking* 1853

#31 food 62475

#32 drink 8985

#33 garden* 1225

#34 eating together 931

#35 meal sharing 52

#36 healthy eating 6736

#37 food intervention 24591

#38 nutrition intervention 26313

#39 18 or 19 or 20 or 21 or 22 or 23 or 24 or 25 or 26 or 27 or 28 or 29 or 30 or 31 or 32 or 33 or 34 or 35 or 36 or 37 or 38 2070101

#40 MeSH descriptor: [Community-Based Participatory Research] explode all trees 332

#41 MeSH descriptor: [Neighborhood Characteristics] explode all trees 5

#42 community based 32382

#43 local 91358

#44 communal 202

#45 collective 1388

#46 public 56186

#47 neighbourhood 1251

#48 neighborhood 1251

#49 community program 21275

#50 community dwelling 6001

#51 40 or 41 or 42 or 43 or 44 or 45 or 46 or 47 or 48 or 49 or 50 876075

#52 17 and 39 and 51 9700

1. **CINAHL**

| **#** | **Query** |
| --- | --- |
| S46 | S15 AND S32 AND S44 |
| S45 | S15 AND S32 AND S44 |
| S44 | S33 OR S34 OR S35 ORS36 OR S37 OR S38 ORS39 OR S40 OR S41 ORS42 OR S43 |
| S43 | neighborhood OR (community services orsupport services orcommunity resources orcommunity organization )OR ( community basedparticipatory research orcbpr or action researchor community engagedresearch ) |
| S42 | "community basedparticipatory research" |
| S41 | "community-dwelling" |

1. **MEDLINE**

|  | **Query** |
| --- | --- |
| S41 | (S30 OR S31 OR S32OR S33 OR S34 OR S35OR S36 OR S37 ORS38) AND (S13 AND S28AND S39) |
| S40 | (S30 OR S31 OR S32OR S33 OR S34 OR S35OR S36 OR S37 ORS38) AND (S13 AND S28AND S39) |
| S39 | S30 OR S31 OR S32 ORS33 OR S34 OR S35 ORS36 OR S37 OR S38 |
| S38 | community basedintervention OR localcommunity ORneighborhood ORcommunity service |
| S37 | "community-dwelling" |
| S36 | (MH "CommunityParticipation") OR"community program" |

1. **SOCINDEX**

| **#** | **Query** |
| --- | --- |
| S11 | S3 AND S6 AND S9 |
| S10 | S3 AND S6 AND S9 |
| S9 | S7 OR S8 |
| S8 | ( community basedparticipatory research orcbpr or action researchor community engagedresearch or local orcommunal or collective orneighbourhood orneighborhood or borough) OR ( public orcommunity ) OR (community program orcommunity service orcommunity dwelling o ) |
| S7 | DE "COMMUNITIES" ORDE "COMMUNITY healthservices" OR DE"COMMUNITYinvolvement" OR DE"COMMUNITY services" |
